# Biased social chromosome transmission in males of the fire ant *Solenopsis invicta*

**DOI:** 10.1093/g3journal/jkae289

**Published:** 2024-12-06

**Authors:** Daniel R Hettesheimer, Haolin Zeng, Brendan G Hunt, Kenneth G Ross

**Affiliations:** Department of Entomology, University of Georgia, 120 Cedar St, Athens 30602, GA, USA; Department of Entomology, University of Georgia, 120 Cedar St, Athens 30602, GA, USA; Odum School of Ecology, University of Georgia, 140 E. Green St., Athens 30602, GA, USA; Department of Entomology, University of Georgia, 120 Cedar St, Athens 30602, GA, USA; Department of Entomology, University of Georgia, 120 Cedar St, Athens 30602, GA, USA

**Keywords:** ant males, diploid males, greenbeard effects, selfish genetic elements, *Solenopsis invicta*, supergene

## Abstract

Selfish genetic elements subvert the normal rules of inheritance to unfairly propagate themselves, often at the expense of other genomic elements and the fitness of individuals carrying them. Social life provides diverse avenues for the propagation of such elements. In the fire ant *Solenopsis invicta*, polymorphic social organization is controlled by a social chromosome, one variant of which (*Sb*) enhances its own transmission in polygyne colonies through effects on caste development and queen acceptance by workers. Whether the selfish effects of *Sb* extend to haploid (reproductive) males in this system is less clear. Here, we demonstrate a strong overrepresentation of the *Sb* social chromosome haplotype in reproductive males, relative to Mendelian expectations, in both the pupal and adult stages. We tested for the presence of selective execution of adult *SB* males by workers but did not detect such behavior. Combined with the presence of a strong imbalance in the haplotype frequencies already early in the pupal stage, these results indicate that the *Sb* supergene may distort male haplotype frequencies during larval or embryonic development. These findings are significant because they demonstrate yet another mode by which the selfish tendencies of the *Sb* supergene are manifested, illuminate complex interactions between *Sb* and the fire ant breeding system, inform the development of models of the population dynamics of *Sb*, and illustrate how a selfish supergene can increase in frequency in a population despite harboring deleterious mutations.

## Introduction

Selfish genetic elements are DNA segments that propagate themselves in a population at the expense of other genes (Ågren and Clark 2018). This is achieved by subverting the normal rules of inheritance, for instance by distorting transmission ratios in their favor during meiosis or by helping conspecifics that bear the element or harming those that lack it. These processes can occur even in cases in which the elements are detrimental to the organisms carrying them (Burt and Trivers 2008). In evolutionary terms, selfish genetic elements are significant due to their ability to greatly influence genome structure and gene frequencies within populations, as well as divergence between them that potentially can culminate in speciation (Werren *et al*. 1988; Hurst *et al*. 1996; Seehausen *et al*. 2014).

Social life provides an opportunity for selfish genetic elements to propagate themselves through effects on social behaviors. In particular, greenbeard effects (Dawkins 1976; Gardner and West 2010; Gardner 2019) are a special situation conceived by Hamilton (1964) where social behaviors are directed toward others based not on their overall genetic relatedness, but instead on their mutual recognition of a shared variant at a single genetic locus. With great foresight, Hamilton noted that “something like a supergene” may be required to produce a greenbeard effect in nature because of the requisite encoded (1) unique, discernable trait, (2) ability of others to perceive this trait, and (3) appropriate social responses (Hamilton 1964). Recently, supergenes composed of chromosomal inversion polymorphisms, which cause multiple protein-coding and regulatory loci to be inherited in tight linkage, have been documented across diverse eukaryotic taxa (Wellenreuther and Bernatchez 2018; Chapuisat 2023). Consistent with Hamilton’s prediction, derived supergene haplotypes acting as selfish genetic elements have been characterized in 2 ant taxa exhibiting independent origins of supergene-mediated alternative social organizations (Chapuisat 2023), including through greenbeard effects in the fire ant *Solenopsis invicta* (Keller and Ross 1998; Trible and Ross 2016).

In polygyne colonies of *S. invicta* (those with multiple reproductive queens), the derived *Sb* supergene haplotype acts selfishly via several documented routes (*Sb* is absent in monogyne colonies; Ross 1997). First, at the onset of development, *Sb* biases its occurrence in eggs in some families, likely via meiotic drive, although expected Mendelian frequencies are recovered at the population level because *Sb* drive appears to be countered by overrepresentation of the alternative supergene variant (*SB*) in other families (Ross and Shoemaker 2018). Second, during larval ontogeny, possession of *Sb* consistently increases the likelihood of female brood developing into queens instead of permanently sterile workers (Buechel *et al*. 2014). Third, in the adult stage, young queens lacking *Sb* (*SB* homozygotes) are subject to aggression and execution, predominantly by workers bearing *Sb* (Keller and Ross 1998). This striking example of a greenbeard effect is mediated by a semiochemical signal exclusive to *Sb*-bearing queens (Trible and Ross 2016; Zeng *et al*. 2022; Zeng 2023). Despite the presence of multiple avenues facilitating propagation of the element, *Sb* incurs fitness costs to individuals in the form of recessive lethal effects in reproductive females (Ross 1997; Hallar *et al*. 2007).

As in most ants, males of fire ants are greatly understudied despite making up half of the breeding pool of individuals, presumably because they play minimal roles in the social life of the colony and can be difficult to observe under natural conditions (Beani *et al*. 2014; Pennell *et al*. 2018). Nonetheless, there are signs that *Sb* may act selfishly in this sex as well as in females, despite the reduced fecundity observed for *Sb* males relative to *SB* males (Lawson *et al*. 2012). Fritz *et al*. (2006) suggested *Sb*-carrying males are overrepresented in polygyne colonies within the invasive US range, a finding that would mirror the greenbeard-mediated overrepresentation of *Sb*-carrying queens. However, the results of Fritz *et al*. (2006) are difficult to integrate into a complete picture of fire ant social and reproductive biology because their experimental design did not permit the differentiation of reproductive haploid males from effectively sterile diploid males (Krieger *et al*. 1999). This is important because of the peculiar pervasiveness of diploid males (>80% of all males) in invasive polygyne *S. invicta* (Ross and Fletcher 1985; Ross *et al*. 1993).

The reasons for the abundance of diploid males in polygyne colonies of *S. invicta* in invasive populations include the male-haploid (arrhenotokous) genetic system characteristic of hymenopterans and the worker-dependent initiation of reproduction characteristic of polygyne ant life histories. In many hymenopterans, the pairing of alleles at a single sex-determining locus is responsible for the fate of fertilized diploid eggs in a process termed complementary sex determination (Whiting 1943; Whiting 1945; Cook 1993). If the egg is fertilized by a male bearing the same complementary sex determination allele as that contributed by the mother, a circumstance resulting from a “matched mating” (Adams *et al*. 1977), there is no complementation (required for development as a female) and the individual develops as an atypical diploid male. Being an invasive species, *S. invicta* experienced an extreme bottleneck while colonizing the U.S., losing allelic diversity at many loci, including the sex-determining locus (Ross and Shoemaker 2008). As a result, matched matings are far more frequent in the United States than the native range (Ross *et al*. 1993). Worker fire ants evidently do not discriminate against diploid males, in contrast to, for instance, honey bees, which destroy diploid male larvae soon after they eclose from the egg (Woyke 1980). Moreover, male diploidy is absent in monogyne *S. invicta* colonies due to extreme selection associated with the claustral mode of colony founding exhibited by this form, in which one to several newly mated queens initiate a colony without foraging or help from workers (Tschinkel and Howard 1983). The burden of producing diploid males at the expense of workers during the claustral period, an unavoidable consequence of matched matings, invariably dooms young match-mated foundresses to failure [when the first workers appear, only one of the foundresses is kept by the colony (Tschinkel 2006)]. In polygyne colonies, most reproductive queens are not match-mated, such that sufficient worker brood is produced to ensure a vigorous and viable colony despite the presence of match-mated queens (Ross and Fletcher 1986).

Our overall goal in this study was to definitively determine whether the *Sb* greenbeard effect extends to reproductive males of *S. invicta*, resulting in the underrepresentation of haploid *SB* males relative to haploid *Sb* males in polygyne colonies. Our first aim was to generate accurate supergene haplotype frequency estimates for the male breeding pool by employing multiple robustly polymorphic genetic markers to assess the individual ploidy of males sampled in our polygyne study population. Such estimates are central to understanding the complex evolutionary dynamics of the *Sb* supergene, as well as being useful for obtaining estimates of inter-form gene flow (between monogyne and polygyne populations). Our second aim was to identify any disparity in haplotype frequencies observed between life stages that may shed light on the ontogeny of a potential greenbeard effect in males. To do so, we characterized haplotype frequencies in pupae as well as adults (males and females are not readily distinguishable at earlier developmental stages). Our final aim was to identify a behavioral mechanism that may explain the overrepresentation of *Sb*, given the relatively equitable transmission of *SB* and *Sb* observed at the embryonic stage (Ross and Shoemaker 2018) and the seemingly higher innate fitness of haploid males bearing *SB* relative to those with *Sb* (Lawson *et al*. 2012). To provide insight on the potential for greenbeard behaviors in this context, we developed an assay to test for discrimination against males by workers. Overall, our results implicate the derived *Sb* supergene haplotype in yet another avenue for biased self-propagation.

## Methods

### Sampling

Whole *S. invicta* nests excavated from the soil at various sites in Athens-Clarke Co., Georgia, U.S. during the spring of 2021 were placed in 19-L buckets. In total, 40 polygyne colonies were collected from the field and returned to our laboratory. Colonies were maintained in a controlled environment rearing room at a temperature of 29–32°C, relative humidity of 40–60%, and a constant photoperiod of 14 h of light followed by 10 h of darkness (Ross 1988). Ants were held in large (54 × 42 cm) plastic trays, the walls of which were coated with an antitraction compound to prevent escape (polytetrafluoroethylene preparation, 60 wt. % dispersion in H_2_O; Millipore Sigma, Burlington, MA). The trays held 14 cm diameter petri dishes containing moistened plaster bottoms that served as nests. Ants were fed several different food mixtures: a high-sugar mixture, a high-protein mixture, and freeze-dried insects homogenized with water in a blender (Zeng *et al*. 2022).

Adult male ants were collected by aspiration from each colony within 3 days of establishment in the laboratory to obtain a representative sample of the natural population of males in polygyne colonies in northern Georgia. Care was taken to collect only mature adults, that is, males with uniformly black cuticle and transparent wings. Once collected, males were frozen (−80°C) in bulk in labeled, screw-cap microcentrifuge tubes corresponding to each colony pending measurement and genetic analysis.

### Distinguishing haploid and diploid males

Our main objective was to obtain unbiased estimates of the social supergene haplotype frequencies for *S. invicta* haploid males from polygyne colonies in the invasive U.S. range, a challenging task because the great majority of polygyne males in this region (80–95%) are diploid (Ross and Fletcher 1985; Ross *et al*. 1993, 1996). Thus, our first goal was to parse out as many diploid males in the bulk collection as early in the pipeline as possible to reduce the potential time and expense of genotyping every male collected. According to Ross and Fletcher (1985), diploid and haploid males comprise 2 distinct but partly overlapping size distributions when comparing area of the mesoscutum [a hard cuticular plate on the dorsum of the mesosoma (thorax)]. Their investigation also suggested that diploid males typically weigh more than their haploid counterparts, although again, weights of the 2 types overlap to some extent. Further complicating matters, haploid males with the *SB* haplotype are significantly larger and heavier than those possessing the *Sb* haplotype (Goodisman *et al*. 1999).

Frozen males chosen for sub-sampling were thawed, blotted dry, then weighed individually to ±0.01 mg. Initially, males were selected haphazardly to create a preliminary weight histogram. This histogram depicted a bimodal distribution, expected to represent haploid (lighter) and diploid (heavier) males. This was confirmed by genotyping males at *Gp-9*, a marker on the *Sb* social supergene of *S. invicta* (Gotzek and Ross 2007; Wang *et al*. 2013), as well as at 4 microsatellite loci, as described below. Multilocus microsatellite genotypes of each weighed male showed that the frequency of haploid males approaches 0 at 8.0 mg, a value in agreement with Ross and Fletcher (1985), who found that 100% of haploid males they collected fell below a weight of 8.4 mg. As a result, we sampled every male below 8.0 mg. During our initial sampling to create the weight histogram, several haploid males heavier than 8.0 mg were found, compelling us to sample subsets of 20 males for every 0.25 mg increment between 8.0 mg and 10.0 mg to ensure that we did not truncate the upper tail of the *SB* weight distribution. Of the 160 additional males genotyped above the 8.0 mg threshold, no haploids were found. The importance of sampling the entire range of different-sized adult haploids is to avoid unintentionally excluding the largest haploid (*SB*) males and thus biasing our haplotype frequency estimate for polygyne males in the wild (Goodisman *et al*. 1999).

Pupal males were collected opportunistically along with the adults. Only white-bodied, pink-eyed pupae were collected in order to eliminate pupal age (and water content) as factors affecting their weight. Because there were relatively few males at this specific point in their ontogeny present in the 40 colonies, all that were collected were weighed and genotyped.

### DNA extraction and Gp-9 genotyping

Gasters (post-petiole abdominal segments) were removed from adult males after weighing, and DNA was extracted from the remainder of the body using the QIAGEN Puregene DNA extraction kit. DNA was extracted from the whole bodies of pupal males.

Extracted DNA was amplified using primers Gp-9_24bS, Gp-9_25bAS, Gp-9_26BS, and Gp-9_16BAS (Valles and Porter 2003). A multiplex PCR was run in 15 μL reactions containing 7.50 μL of Taq DNA polymerase (TaKaRa Premier Ex Taq HS, Cat# RR030), 0.15 μL each of Gp-9_24bS and Gp-9_25bAS primers at 50 μM, 0.30 μL each of Gp-9_26BS and Gp-9_16BAS primers at 50 μM, 4.50 μL of water, and 2 μL of DNA template (diluted 1:4 in water) (modified from Valles and Porter 2003). The reactions took place in a BIO-RAD T100 thermal cycler using the following program: 94°C, 2 min; 34x (94°C, 15 s; 55°C, 15 s; 68°C, 30 s); 68°C, 5 min; 10°C until terminated. The *Gp-9* genotype (haplotype) of each male was scored after running the PCR product out on a 1.5% agarose gel stained with ethidium bromide. The PCR product of the *B* allele of *Gp-9* (*SB* supergene haplotype) using these primers is 517 bp, while the product of the *b* allele (*Sb* supergene haplotype) is 423 bp (Valles and Porter 2003).

### Microsatellite genotyping

Microsatellite genotyping was conducted using adult and pupal male template DNA to distinguish diploid homozygous from haploid hemizygous males, both of which yield single bands (PCR products) on gels in the *Gp-9* PCR assay. Male DNA served as templates for genotyping at 4 amply polymorphic microsatellite loci: *Sol42_f, Sol49, C536,* and *cassidy* (Ascunce *et al*. 2009) (Supplementary Table 1). PCRs were performed using primers Sol-42_for_VIC, Sol-42_rev, Sol-49_for_Fam, Sol-49_rev, Sdag-C536-F_PET, Sdag-C536-R, cassidy_F_VIC, and cassidy_Rp. Amplification was conducted in a BIO-RAD Peltier Thermal Cycler-100 under the following program: 94°C, 90 s; 60°C, 45 s, −5°C per cycle; 72°C for 1 min; repeat cycle 9 times. 94°C, 30 s, 55°C, 45 s; 72°C, 1 min; repeat 24 times. 72°C, 1 min; 10°C until terminated. After the PCR was completed, the products were sequenced by the commercial service GENEWIZ (Azenta Life Sciences, Burlington, MA). Chromatograms were scored with the aid of Thermo Fisher’s online cloud fragment analysis tool. Given the number and variability of the microsatellite markers (Supplementary Table 1), we estimate that among the males with single PCR products (bands) in the *Gp-9* assay, the probability of classifying a diploid homozygote at this gene falsely as a haploid hemizygote using the 4 microsatellites is 0.5%.

### Correcting for social structure

There are a number of factors linked to social systems that have the potential to influence the results of population surveys. In our case, we wished to consider factors that vary between colonies, such as colony-level effects on weight (due to any of several potential factors that vary between nests), genetic nonindependence of samples (male nestmate relatedness >0), and uneven sampling of colonies, all of which can potentially skew the weight distributions or haplotype frequencies that we generated.

**Fig. 1.**
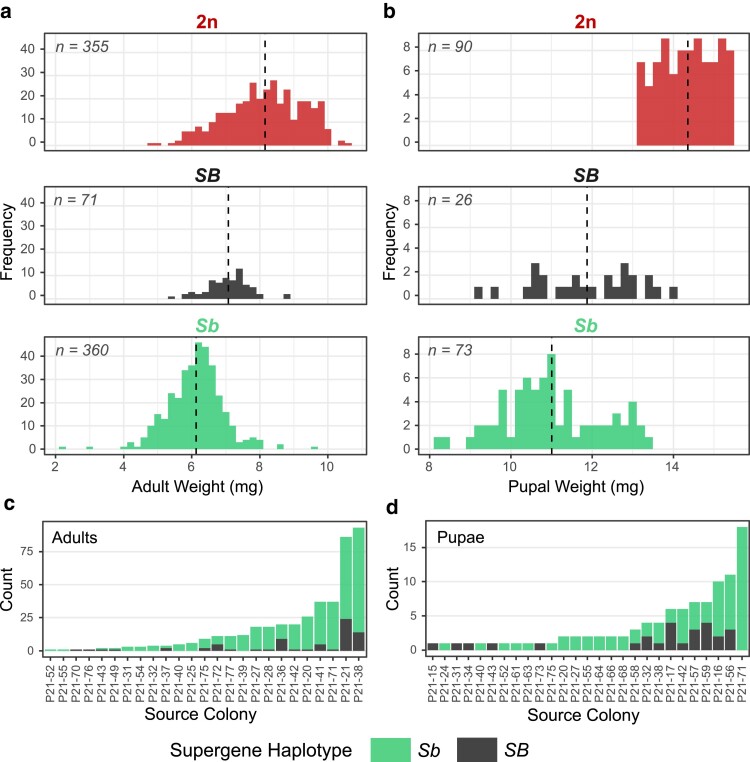
Male weight and ploidy in the polygyne form of invasive *S. invicta*. Upper panels display male weight and supergene genotype for adults a) and pupae b). Dashed lines indicate the mean male weight in each panel. The adult diploid (2*n*) histogram represents a small fraction of the individuals originally collected due to our exclusion of the heaviest males and so is not representative of the frequency or weight of adult diploid males in the wild. The lower panels show the frequencies of haploid male adults c) and pupae d) in male-producing colonies.

To account for genetic nonindependence, we first calculated the genetic relatedness between all pairs of nestmate pupal and adult haploid males using the program PolyRelatedness V1.11b (Huang *et al*. 2015). The same microsatellite genotypic data used to determine ploidy of males were used to estimate haploid male nestmate relatedness, with the reference (base) population allele frequencies obtained from a sample of 172 wingless (reproductive) polygyne queens and 166 of their male mates from 22 nests collected in the same area where the study males were collected (see Weir *et al*. 2006; Wang 2014 for discussions of the importance of the reference population). To visualize the potential importance of uneven sampling, we generated histograms depicting the numbers of samples of haploid pupal and adult males of each haplotype that we obtained from each of the 40 study colonies (Fig. 1, c and d).

To account for the effects of genetic nonindependence and uneven sampling on our haplotype frequencies, we employed a resampling approach that weighted colonies equally (rather than individual males) while making full use of our data. We selected one male per colony at random to calculate population haplotype frequencies, then repeated this process (with replacement) 499 times. The 500 resampled values were averaged to estimate population haplotype frequencies, with the 2.5th and 97.5th percentile values taken to bound the 95% confidence intervals (CIs). We repeated this resampling method with individual weights of males to estimate unbiased population-wide weight averages and one-tailed confidence limits for each haplotype.

### Male execution assay

We performed an assay to monitor the fate of adult males of both supergene haplotypes in the presence of polygyne workers. We targeted small to medium-sized males in order to perform the assay with as many haploids and as few diploids as possible. Adult males were sampled from newly collected polygyne colonies in fall 2023, weighed, then individually isolated inside 0.6 mL PCR tubes. A small hole in the bottom of each tube allowed workers to move freely into and out of the tube. Holes were made large enough to allow even major workers to pass in and out of the tube but not so large that males could fit through them.

Males placed inside the tubes were immediately returned to their colonies of origin by placing the tubes in the large foraging area of the tray outside of the nest (Supplementary Video 1). Worker/male interactions were monitored once every hour for the next 12 h to determine how the males were treated by nestmate workers. If workers were observed biting or stinging a male, we removed the tube and scored the trial outcome as an execution. We also removed tubes with intact or partially dismembered dead males that had been executed during the time between observation points. Attacked/executed males as well as males that survived until the end of the 12-h observation window were frozen for subsequent DNA extraction and genotyping at *Gp-9* and the 4 microsatellites. We ran an additional execution assay using monogyne males in monogyne assay colonies to learn if our laboratory rearing conditions invariably provoke execution of males and whether there are general differences between the social forms in their tolerance of adult males.

## Results

### Effect of supergene haplotype on male weight

We genotyped a total of 790 adult males and 188 pupal males at *Gp-9*; of these, 478 adults and 113 pupae with single stained bands (PCR products) in the assay gels, the ploidy of which was uncertain, were genotyped at the 4 microsatellite loci. The resulting 530 confirmed adult and pupal haploid males’ weight distributions are depicted by haplotype in Fig. 1, as are those of the remaining sampled (diploid) males.

Adult haploid males possessing the *Sb* supergene weigh significantly less than *SB* males; on average, *Sb* males weighed 6.12 mg while *SB* males weighed 7.07 mg (*P* < 0.0001, *t*-test). This result is expected, as Goodisman *et al*. (1999) and Lawson *et al*. (2012) reported larger size and higher weight, respectively, of *SB* males compared with *Sb* males. After correcting for genetic nonindependence and uneven sample sizes per colony by resampling, the disparity between haplotype weight averages remained significant; on average, *Sb* males weighed 6.36 mg, and *SB* males weighed 6.96 mg (*P* < 0.00001 based on the confidence limits) using our resampling approach. It should be mentioned that because we avoided sampling the largest males in this study, we cannot accurately estimate the average weight of adult diploid males. However, even among the males in our truncated sample, the average diploid male weighed significantly more than the average haploid male [8.15 mg vs. 6.28 mg, (*P* < 0.0001, *t*-test)] (Fig. 1). Results for the weights of male pupae closely mirrored those for the adults (Fig. 1).

### Supergene haplotype frequencies in haploid males of the polygyne form

Among the confirmed haploid males sampled from polygyne colonies in our study, those carrying the *SB* variant at the supergene are greatly underrepresented while those carrying *Sb* are greatly overrepresented (Table 1). In the adult stage, the observed uncorrected haplotype frequencies are less than 17% *SB* and greater than 83% *Sb*, far from the 50:50 ratio expected (*P* < 0.0001, binomial test) given that every polygyne reproductive queen in the invasive range is an *SB/Sb* heterozygote and fertile males are impaternate (they represent the products of unfertilized haploid eggs). A similar but slightly less extreme imbalance in the supergene haplotype frequencies is seen in the pupal stage (Table 1); the sampled population of haploid pupae consists of fewer than 27% *SB* males and over 73% *Sb* males, frequencies that again differ significantly from 0.5 (*P* = 0.0001, binomial test). The differences in adult and pupal haplotype frequencies, although modest, are statistically significant (*P* = 0.030, Fisher’s exact test).

**Table 1.** Estimated male haplotype frequencies at the social supergene in the polygyne form of invasive *S. invicta*.

	Uncorrected data*^a^*		Resampled data*^a^*	
	*SB*	*Sb*	*SB*	*Sb*
**Adults**	0.167 (0.136, 0.200)	0.833 (0.798, 0.866)	0.218 (0.214, 0.224)	0.782 (0.776, 0.787)
**Pupae**	0.263 (0.180, 0.350)	0.737 (0.646, 0.818)	0.314 (0.310, 0.319)	0.686 (0.681, 0.690)

^*a*^Both uncorrected and resampled frequencies are listed with 95% CIs in parentheses. CIs for the uncorrected data were estimated by bootstrapping the proportion using 5,000 replicates, while those for the resampled data were obtained as described in the main text.

The haplotype frequencies corrected for nonindependence of the genetic data and uneven sample sizes per colony (see Fig. 1) by resampling the data are slightly less extreme than the uncorrected frequencies, but again are highly significantly different from 50%, judging from the 95% CIs (Table 1).

To clarify the relative importance of colony of origin on weight variation in adult haploid males, we conducted a two-way ANOVA. Both colony of origin (*F* = 9.07, df = 16, *P* < 2e−16) and supergene haplotype of a male (*F* = 121.45, df = 1, *P* < 2e−16) were highly significant factors influencing weight, while the interaction term was nonsignificant. Notably, of the total of 8 haploid males heavier than 8.0 mg we discovered, 4 were from the same colony, an observation consistent with the presence of strong colony-level effects on male weight. This justifies our resampling approach to calculating the average weights of adult males with the alternate haplotypes.

We estimated the average genetic relatedness of haploid male nestmates to be 0.198 (95% CIs: 0.192, 0.205) for adults and 0.169 (0.109, 0.220) for pupae, with the pupal estimate significantly lower than the adult estimate (bootstrapping difference test using single-colony values; 5000 iterations). Considering that brothers have a pedigree-based relatedness of 0.5 with a male-haploid genetic system, our estimates illustrate that at least some haploid male nestmates are quite close kin. Indeed, using equation (2) from Kümmerli and Keller (2007) and assuming relatedness of 0 between nestmate queens and between their male mates (Ross *et al*. 1993), we estimate that effectively 2.5–3.0 queens per colony can explain the genetic diversity of nestmate haploid males included in this study, emphasizing again their genetic nonindependence.

It is apparent that different polygyne colonies produce vastly different numbers of haploid males (Fig. 1, c and d). In 15 of the 40 colonies obtained for this study, no haploid males were present, despite a mean of 10.8 haploid males obtained per colony. The 2 largest colonies accounted for 41.5% of the total haploid males sampled in the study. This highly uneven sampling likely explains most of the differences between the uncorrected and resampled allele frequency and average weight estimates.

### Male execution assay

**Fig. 2.**
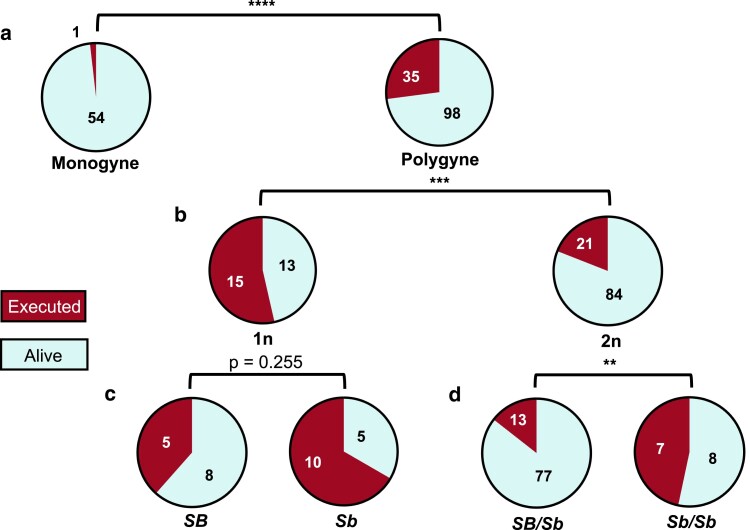
Adult male execution by workers. a) Male execution rates in monogyne vs. polygyne colonies. b) Male execution rates for haploid vs. diploid males within polygyne colonies. c) Haploid male execution rates for the 2 supergene haplotypes within polygyne colonies. d) Diploid male execution rates for 2 supergene genotypes within polygyne colonies. ****P < 0.0001, ***P < 0.001, **P < 0.01; Fisher’s exact tests.

Results from our male execution assay showed that polygyne workers executed more than half of haploid males presented to them, while monogyne workers executed virtually none (*P* < 0.0001, Fisher’s exact test) (Fig. 2). Among the polygyne haploid males, polygyne workers did not execute *SB* and *Sb* males at significantly different rates (*P* = 0.255, Fisher’s exact test; *SB*: *n* = 13, *Sb*: *n* = 15). Thus, our male execution data do not reveal strong discrimination by polygyne workers against adult males lacking the *Sb* variant of the supergene. Unexpectedly, we found that polygyne workers executed a significantly higher proportion of haploid males [0.538 (95% CIs 0.357, 0.714)] than diploid males (0.198 [0.123, 0.274]) (*P* = 0.0007, Fisher’s exact test). In addition, polygyne workers executed diploid males with the *Sb/Sb* genotype [0.469 (0.200, 0.733)] at the social supergene at a significantly higher rate than those possessing the *SB/Sb* genotype [0.144 (0.078, 0.222)] (*P* = 0.008, Fisher's exact test) (*SB/SB* males were too rare for statistical comparison with other diploid males). The absence of discrimination between adult haploid males based on their supergene haplotype, together with the quite similar haplotype frequencies in pupal and adult haploid males, suggest that most of the haplotype frequency imbalance observed in both stages is likely to be imparted at earlier developmental stages. The lack of aggression against males noted in monogyne colonies indicates that male execution in the polygyne colonies is not an artifact of some feature of the artificial rearing environment.

## Discussion

Earlier studies of the genotype and allele frequencies in the different sexes and life stages of polygyne colonies of the fire ant *S. invicta* have been instrumental in inferring how the *Sb* supergene in this ant perpetuates itself and spreads within and between the social forms via the fundamental evolutionary forces of selection, gene flow, and transmission distortion (e.g. Ross 1997; Ross and Shoemaker 1997, 2018  DeHeer *et al*. 1999; Goodisman *et al*. 2000; DeHeer 2002; Buechel *et al*. 2014). The haplotype frequencies we estimated suggest the *Sb* allele acts selfishly by increasing its frequency among fertile males, as in females, the effects of which are evident in both the pupal and adult stages. The underrepresentation of *SB* males in both life stages accords with the conclusions reached by Fritz *et al*. (2006) after surveying males in a polygyne population in Florida, U.S. without distinguishing the prevailing sterile diploid males from fertile haploid males. The fact that *SB/SB* diploid males were too rare for inclusion in our execution assay, along with the observations of Fritz *et al*. (2006) based primarily on diploid males, suggest that *SB/SB* males may be present at even lower frequencies than haploid *SB* males in polygyne colonies, although the significance of such a pattern is unclear. We hypothesize that most of the imbalance we document for haploid males arises during the larval stages because population haplotype frequencies appear to be relatively equal in the pool of eggs laid by polygyne queens (Ross and Shoemaker 2018). Our results thus add to a growing list of examples of selfish behaviors and developmental outcomes induced by supergenes in social systems, illustrating the diversity of routes by which selfish elements can operate to bias their representation in succeeding generations to their advantage (Chapuisat 2023).

The relatively small disparity between adult and pupal haplotype frequencies we report is likely a sign of continuing selection that takes place sometime between the early pupal and mature adult stages. This may involve a greenbeard signal associated with the *Sb* genotype (likely a cuticular compound; Zeng *et al*. 2022) that becomes more conspicuous to workers as males mature as adults, a phenomenon observed in queens (Keller and Ross 1998). However, this does not explain the mechanism by which the relatively steep decline in *SB* male frequency in larvae is achieved, for instance by execution or neglect. A second potential factor contributing to the decline in *SB* larvae could be intrinsic ontological differences between *SB* and *Sb* males, such as growth rate or viability. Lastly, perhaps *SB* males of *S. invicta* experience a fate analogous to that of some eggs laid by heterozygous polygyne queens of the ant *Formica selysi*, which, like *S. invicta*, has an inversion-based supergene that controls colony queen number (Table 2). Eggs lacking the polygyny-inducing variant of the *F. selysi* supergene undergo arrested development and fail to hatch, thus biasing the transmission ratio in favor of the alternate supergene haplotype (Avril *et al*. 2020).

**Table 2.** Comparison of supergene effects on transmission and reproductive fitness in the ants *Solenopsis invicta* and *Formica selysi* (diverged around 100 mya).

	*S. invicta–Sb* supergene	*F. selysi–Sp* supergene
**Supergene characteristics**	Young, large *Sb* (vs. *SB*) supergene caused by 3 inversions (origins ∼0.5 mya; >10 Mb) (Yan *et al*. 2020), spread via introgression to multiple *Solenopsis* species (Stolle *et al*. 2022; Helleu *et al*. 2022), with greatly expanded size for *Sb* vs. *SB* (Stolle *et al*. 2019)	Old, large *Sp* (vs. *Sm*) supergene caused by multiple inversions (origins >20 mya; >10 Mb), shared by multiple *Formica* species (Purcell *et al*. 2014; Brelsford *et al*. 2020)
**Monogyne colony genotypes**	*SB/SB*, *SB* (Ross 1997; Wang *et al*. 2013)	*Sm/Sm*, *Sm* (Purcell *et al*. 2014)
**Polygyne colony genotypes**	*SB/Sb* (rarely *Sb/Sb*) reproductive queens; *SB/SB*, *SB/Sb*, *Sb/Sb*, *SB*, *Sb* offspring (Ross 1997; Wang *et al*. 2013)	*Sm*/*Sp* & *Sp/Sp* reproductive queens; *Sm/Sp*, *Sp/Sp*, *Sp* offspring (Purcell *et al*. 2014)
**Avenues for biased transmission**	Greenbeard discrimination against *SB/SB* mature gynes and queens by workers (Keller and Ross 1998; Gotzek and Ross 2008); biased queen development for *SB/Sb* vs. *SB/SB* (Buechel *et al*. 2014); underrepresentation of *SB* among haploid males (this study)	Maternal-effect killing (failure to hatch) of eggs lacking *Sp* haplotype prevents transmission of *Sm* homozygotes and hemizygotes in polygyne colonies (Avril *et al*. 2020)
**Effects on queen traits**	Recessive deleterious effects observed for *Sb* (Hallar *et al*. 2007); *Sb*-carrying queens accumulate less fat reserves and have lower dispersal ability than *SB/SB* queens (DeHeer *et al*. 1999; Shoemaker *et al*. 2006); lower fecundity and colony founding ability for *Sb*-carrying vs. *SB/SB* queens (Vargo and Fletcher 1989; DeHeer 2002)	Recessive deleterious effects observed for *Sp* (Blacher *et al*. 2023); *Sp*-carrying queens accumulate less fat reserves and have lower dispersal ability than *Sm/Sm* queens (De Gasperin *et al*. 2024); lower fecundity and colony founding ability observed for *Sp*-carrying vs. *Sm/Sm* queens (Reber *et al*. 2010; Blacher *et al*. 2023)
**Effects on male traits**	*Sb* males are smaller and have lower sperm counts than *SB* males (Lawson *et al*. 2012)	*Sp* males are smaller and have lower sperm counts than *Sm* males (De Gasperin *et al*. 2024)

The logical next step in clarifying the source of the fire ant male bias would be to examine embryos and each larval instar for departure from a 50:50 ratio of the haplotypes of lesser or equal magnitude to that seen in the pupae. Unfortunately, male larvae are indistinguishable from worker or queen larvae by appearance alone, so intense sampling and genotyping (or other technical innovations) would be needed to filter down to even a modest sample of haploid male larvae. Because of this limitation, we did not sample male larvae to detect haplotype skew during larval development, the stage at which we propose the distortion between *Sb* and *SB* frequencies arises. We also were unable to evaluate survival of embryos to test for a potential contribution of maternal effects on prelarval viability.

The difference in weight we observed between *SB* and *Sb* males appears to reflect more limited dispersal capabilities and reduced reproductive capacity for sexuals carrying the polygyne-affiliated (*Sb*) variant (Lawson *et al*. 2012), as observed in queens (DeHeer *et al*. 1999; Krieger *et al*. 1999; DeHeer 2002). Such genetic associations with limited dispersal and fecundity in both sexes are also apparent in the independently evolved supergene system underlying the polygyny syndrome in *F. selysi* (Table 2) (Chapuisat 2023; De Gasperin *et al*. 2024). The existence of distinct colony founding life histories associated with the genesis of monogyne and polygyne colonies within a species allows antagonistic selection to operate within reproductive castes (Keller and Passera 1989; Rüppell and Heinze 1999), while supergene systems underpinning social polymorphisms provide a genetic architecture favorable to resolution of such evolutionary antagonism (Avril *et al*. 2019; Fontcuberta *et al*. 2021; Zahnd *et al*. 2021; De Gasperin *et al*. 2024).

We were unable to determine the proximate cause of the modest increase in haplotype skew from the pupal to the adult stage in our execution assay designed to do so—no statistically significant difference in execution rates occurred between adult males with each haplotype. We expected to observe some bias against *SB* males by polygyne workers analogous to the well-documented phenomenon of such workers executing young *SB/SB* adult queens (Keller and Ross 1998, 1999). However, failure to detect a difference is not surprising, considering that most of the haplotype skew originates prior to the adult stage.

Most unexpected, however, was the disparity between execution rates of haploid vs. diploid males in polygyne colonies, as workers killed diploids significantly less frequently than haploids. We speculate that this may be a result of physiological feminization of diploid males throughout development, a common phenomenon in diverse taxa with haplo-diploid sex determination (Smith and Wallace 1971; Duchateau and Mariën 1995; Giorgini *et al*. 2009; Hitchcock *et al*. 2022). In diploid males of *S. invicta* this appears to include formation of an incomplete reproductive tract Hung *et al*. 1974; Krieger *et al*. 1999) and developmental gene expression and DNA methylation patterns similar to those of diploid females (Glastad *et al*. 2014; Nipitwattanaphon *et al*. 2014). The difference in execution rates between *SB/Sb* and *Sb/Sb* diploid males is difficult to explain but may contribute to known recessive deleterious effects of *Sb* (DeHeer 2002; Hallar *et al*. 2007; Lawson *et al*. 2012). The lack of male execution in our monogyne control colonies may indicate that this represents some real biological phenomenon differing between the 2 social forms likely stemming from the presence of *SB/Sb* workers.

As important as demonstrating another selfish property of the *Sb* element, our results contribute to an emerging picture of complex, cascading interactions between such elements, the breeding system, and colony social structure in ants (Table 2) (Lawson *et al*. 2012; Avril *et al*. 2019). The favored production of *Sb* over *SB* males by polygyne colonies is, in this scenario, assumed to translate into a relatively elevated frequency of *Sb* males in the pool of potential mates of polygyne queens during mating flights [other factors, including proximity of monogyne colonies, also affect composition of this pool (Ross and Keller 1995; Fritz *et al*. 2006)]. The hypothesized result is a larger proportion of polygyne queens mating with *Sb* males than would be the case if polygyne males were produced in the expected 50:50 ratio of the 2 haplotypes. However, males with *Sb* also produce, on average, one-third fewer sperm cells than *SB* males (Lawson *et al*. 2012) and virtually all polygyne queens that mate with an *Sb* male are polyandrous, while the vast majority that mate with an *SB* male remain monandrous (Lawson *et al*. 2012). Thus, the male-mediated effects of the *Sb* haplotype on the breeding system of fire ants are complex and, like the supergene system of *F. selysi* (Avril *et al*. 2019; De Gasperin *et al*. 2024), feedback to influence queen polyandry and colony kin structure in ways that further prescribe arenas for potential conflict among nestmates. Despite these complexities, our quantification of *Sb* haplotype frequencies in fertile polygyne males will contribute to improved models of the dynamics of *Sb* transmission and spread based on empirical frequency estimates across life stages, sexes, and castes (e.g. Ross 1992, Ross 1997; Ross and Shoemaker 1993; Goodisman *et al*. 2000).

To conclude, our findings add to a growing knowledge base on the importance of the social supergene to the social and breeding biology of polygyne *S. invicta*. We now know that the *Sb* supergene variant exerts a unique evolutionary force in *S. invicta* by strongly skewing haplotype frequencies in functional males in its favor despite the inherent fitness disadvantages to individuals bearing it. Associating deleterious traits with the biased transmission properties of the *Sb* supergene variant is important for demonstrating the effectiveness of the supergene in driving social evolution in *S. invicta*, as well as for understanding how, in general, a supergene can increase in frequency despite harboring deleterious mutations that would otherwise hinder the spread of such elements. The route suggested by the bias against *SB* males we observed is just one of many that the *S. invicta* supergene uses to cheat within the unique polygyne fire ant social environment.

## Supplementary Material

jkae289_Supplementary_Data

## Data Availability

The raw data for this study (weights, *Gp-9* and microsatellite haplotypes/genotypes of males) are included as a Supplementary data file. Supplementary Video 1 is available at GSA FigShare: https://doi.org/10.25387/g3.27956325. Supplemental material available at G3 online.
