# Supplementary material for: Biased social chromosome transmission in males of the fire ant Solenopsis invicta
Source: G3 (Bethesda). 2024 Dec 6;15(2):jkae289. doi: 10.1093/g3journal/jkae289 (PMC11797023; doi:10.1093/g3journal/jkae289)
Supplement: jkae289_Supplementary_Data [file jkae289_supplementary_data.zip › Supplementary_Material_G3-2024-405556.pdf]

Supplementary Materials

Biased social chromosome transmission in males of the fire ant *Solenopsis invicta*

Table S1. Characteristics of microsatellite loci used in this study. Shown in the table are various statistics and the chromosomal location for four microsatellite loci used to differentiate hemizygote haploid males from homozygote diploid males at the gene *Gp-9*.

Locus	Count of alleles observed	Expected heterozygosity	Estimated prob. of null allele†	Chromosome
Sol_42f	16	0.388	0.001	15
Sol_49	8	0.907	0	8
C536‡	3	0.328	0.018	6
cassidy	5	0.654	0	7

† Based on 9287 female pupae from the same population as males in this study (Hale Walker et al. in preparation) using method of Kalinowski and Taper (2006).

‡ The primer set for locus *C536* and another microsatellite marker, *C27*, are transposed in the original publication describing them (Ascunce et al. 2009).

Supplementary Data File

Weight and genotype data of haploid and diploid male *S. invicta* are reported in SupplementaryDataFile.xlsx

Supplementary Video

Video S1 is hosted on GSA FigShare.

Supplementary References

Ascunce MS, Bouwma AM, Shoemaker D. 2009. Characterization of 24 microsatellite markers in 11 species of fire ants in the genus *Solenopsis* (Hymenoptera: Formicidae). *Mol Ecol Res* 9:1475-1479.

Kalinowski ST, Taper ML. 2006. Maximum likelihood estimation of the frequency of null alleles at microsatellite loci. *Cons Gen* 7:991-995.

Hale Walker S, Lacy KD, Ross KG, Zeng H. in preparation. A comprehensive account of the breeding systems of the model ant *Solenopsis invicta*.
